# Impact of HIV co-infection on liver fibrosis regression after HCV treatment

**DOI:** 10.1590/S1678-9946202567080

**Published:** 2025-11-14

**Authors:** Ferdinando Lima de Menezes, Vivian Iida Avelino-Silva, Paulo Roberto Abrão Ferreira

**Affiliations:** 1 Universidade Federal de São Paulo, Departamento de Doenças Infecciosas e Parasitárias, São Paulo, São Paulo, Brazil; 2 Vitalant Research Institute, San Francisco, California, USA; 3 University of California San Francisco, Department of Epidemiology and Biostatistics, San Francisco, California, USA; 4 Universidade de São Paulo, Faculdade de Medicina, Departamento de Moléstias Infecciosas e Parasitárias, São Paulo, São Paulo, Brazil

**Keywords:** Chronic hepatitis C, Liver fibrosis, Elastography, HIV co-infection, Direct acting antivirals

## Abstract

Chronic hepatitis C virus (HCV) infection is a major cause of liver cirrhosis and hepatocellular carcinoma, which may lead to liver transplantation. Co-infection with HIV may accelerate liver disease and impact treatment response. Monitoring liver fibrosis involves non-invasive methods such as transient hepatic elastography (THE), AST to Platelet Ratio Index (APRI), and Fibrosis-4 (FIB-4). This study compared changes in THE, APRI, and FIB-4 among patients with HCV alone and those with HIV-HCV co-infection before and after direct-acting antiviral (DAA) therapy. We conducted a retrospective cohort study using medical records from patients treated at a reference clinic in Sao Paulo, Brazil, between January 2015 and February 2019. Fibrosis assessments (THE, APRI, FIB-4) were performed pre-treatment and six months post-treatment. APRI and FIB-4 were also evaluated at 12 months. Among 148 participants, 105 (70%) had HCV mono-infection and 43 (30%) had HIV-HCV co-infection. Genotype 1 was most prevalent (86%). At six months post-treatment, greater reductions in THE, APRI, and FIB-4 were observed in the HCV mono-infection group. Pre-treatment THE values positively correlated with subsequent reductions. However, multivariable analysis showed no significant differences between groups in THE reductions, and no significant group differences in APRI or FIB-4 at six and 12 months. DAA treatment led to fibrosis regression in most participants. HIV co-infection did not significantly alter fibrosis outcomes following successful HCV treatment.

## INTRODUCTION

Chronic hepatitis C virus infection (HCV) is the leading cause of liver cirrhosis and hepatocellular carcinoma (HCC), and the primary indication for liver transplantation worldwide^1 , 2^ . The natural history and outcomes of HCV infection are largely aggravated by co-infection with HIV^3 , 4^ , with accelerated liver fibrosis, earlier cirrhosis onset, and a higher risk of decompensated liver disease compared to HCV infection alone^5^ . Moreover, when comparing with only HIV infection, it is estimated that HIV-HCV co-infection reduces life expectancy, on average, by 17.3 years^6 , 7^ .

HCV treatment has undergone significant changes in recent years, transitioning from interferon (IFN)-based regimens to direct acting antivirals (DAAs), which have become the standard of care for HCV infection treatment. DAAs offer improved safety, tolerability, and high rates of sustained viral response (SVR) in both HCV infection and HIV/HCV co-infection^8 , 9^ .

Besides eliminating the virus, treatments using DAAs are also related to reducing liver fibrosis^10 – 12^ . However, there remains a scarcity of real-life data on this outcome comparing treatment on HCV infection and HIV-HCV infection^13 , 14^ .

Currently, non-invasive methods to quantify liver fibrosis include the transient hepatic elastography (THE)^15 – 19^ and biomarker-based scoring assessments such as the AST to Platelet Ratio Index (APRI) and the Fibrosis-4 (FIB-4) score^20 – 22^ . Our study aims to evaluate the dynamics of biomarker values and THE before and after SVR comparing participants with HCV infection to participants with HIV-HCV co-infection.

## MATERIALS AND METHODS

### Study design and setting

This retrospective cohort study was at the Infectious Diseases Outpatient Clinic of Hospital Sao Paulo – Federal University of Sao Paulo, UNIFESP, a public, tertiary service which provides care for approximately 20 thousand people yearly in Sao Paulo State, Brazil.

### Ethical aspects

The study protocol was reviewed and approved by the institutional ethics committee (approval N° 2.469.148), including exemption of informed consent for the subset of participants who could not provide consent for participation. We maintained all identifiable information of the participants confidential throughout the study. Individual participant consent was requested from those who were still being followed for HCV treatment. The institutional review board allowed clinical data to be used from clinic patients who could not be contacted for study participation, or whose treatment was completed.

### Study population

We identified all participants ≥ 18 years of age with a diagnosis of chronic HCV infection with SVR following treatment with DAAs between January 2015 and February 2019. HCV infection was confirmed by Anti-HCV serology and quantitative PCR for HCV RNA, further characterized with viral genotyping examination. For participants with HIV co-infection, those who were adherent to antiretroviral treatment, had undetectable HIV viral load and had no active AIDS-defining illnesses were considered eligible.

Every participant was screened for other causes of liver dysfunction such as excessive alcohol consumption (≥ 40 g/day for women and ≥ 60 g/day for men), autoimmune hepatitis, co-infection with hepatitis B virus, Wilson's disease, hereditary hemochromatosis, and systemic infections. They were also screened for conditions that pose a risk of poor adherence, such as severe pre-existing psychiatric disorders and illicit drug use in the 12 months prior to assessment for inclusion. No participants met these criteria, and no one was excluded for these reasons.

### Study procedures and measurements

We extracted all demographic and clinical information used in the analysis from medical charts using a standardized data collection form. The THE was performed by an experienced specialist using the Fibroscan® 502 F01088, with results reported in kilopascals (kPa). AST and ALT measurements, and platelet counts for the calculation of APRI and FIB4 were performed using the enzymatic method (AST and ALT) and the automated complete blood count (platelets) in routine exams. The APRI was calculated as AST/40(ULN)/(Platelets/1000)*100^23^ . FIB4 scores were calculated as (Age*AST)/((Platelets/1000)*)^24^ .

Using the APRI scores, we categorized liver fibrosis into three levels (> 1.5; 0.51–1.50; and ≤ 0.5). Similarly, we categorized participants into three groups according to FIB-4 scores (> 3.25; 1.46–3.25; and ≤ 1.45). Fibrosis stages according to the THE were as follows: F0, 0 to 5.4 kPa; F1, 5.5 to 7.0 kPa; F2, 7.1 to 9.4 kPa; F3, 9.5 to 12.4 kPa; and F4, > 12.5 kPa.

APRI and FIB-4 were evaluated prior to treatment initiation, at six months post-treatment, and 12 months post-treatment. The THE was performed for all participants before the onset of treatment with DAAs and at six months after treatment completion. The change in fibrosis levels was calculated for each participant as the difference between pre-treatment and six months post-treatment measurements in APRI, FIB-4, and THE.

### Statistical analysis

The number of participants was determined by the number of patients seen in clinic. We describe the characteristics of the participants by group using counts, percentages, medians, and interquartile ranges. Group comparisons were done using the Pearson's chi-squared or Fisher's exact test as appropriate for categorical variables, and the Wilcoxon Mann-Whitney rank-sum test for numeric variables. We used univariable and multivariable linear regression models with robust standard errors to investigate the association between co-infection with HIV and change in the THE score with and without adjustment for age, sex, and baseline THE. Age was categorized into quartiles for the regression models. All tests were two-tailed, and p-values < 0.05 were considered statistically significant. The analyses were conducted using Stata 15.1 (StataCorp College Station, TX: StataCorp LP).

## RESULTS

### Characteristics of the study population

We initially identified 256 participants diagnosed with chronic HCV infection who received treatment with DAAs between January 2015 and February 2019 in our clinic. We excluded 23 who were being followed at other clinics and therefore complete tests related to fibrosis were unavailable for review; 77 did not complete the THE exam post treatment, despite being contacted multiple times and eight people failed to achieve SVR. Our final sample included 148 participants, and they were divided into two groups depending on HIV status with 105 in the HCV infection group and 43 in the HIV-HCV co-infection group.

Table 1 describes the characteristics of study participants and genotype 1 was the predominant for both groups. Most participants in the HCV infection group were females (62%), with a median age of 61 years, and only 7% were classified as F0 or F1 based on the pre-treatment THE. In contrast, in the HIV-HCV co-infection group, most participants were males (79%) with a median age of 53 years, and 39% had F0 or F1 on the pre-treatment THE; the median pre-treatment THE score was significantly lower in this group compared to the HCV infection group. Groups were not significantly different regarding the remaining characteristics, including APRI and FIB-4 scores. Table 1 also describes information regarding treatment.

**Table 1:** Characteristics of study participants by group at baseline

		HIV-HCV N = 43	HCV N = 105	p-value
Sex (%)				< 0.001
	Male	34 (79)	40 (38)	
	Female	9 (21)	65 (62)	
Median age, years (IQR)		53 (48–57)	61 (51-–9)	< 0.001
Genotype (%)				
	1	37 (86)	90 (87)	0.827
	Other	6 (14)	13 (13)	
Prior treatment with Peg-IFN (%)		20 (47)	32 (30)	0.064
Median baseline THE (IQR)		9.2 (5.6–18.2)	11.4 (8.6–16.9)	0.042
THE categories (%)				
	F0	8 (18)	3 (3)	
	F1	9 (21)	4 (4)	< 0.001
	F2	5 (12)	22 (21)	
	F3	5 (12)	27 (26)	
	F4	16 (37)	49 (46)	
Median APRI score (IQR)		0.65 (0.36–1.14)	0.64 (0.44–1.09)	0.982
APRI level (%)				
	≤ 0.50	17 (39)	33 (34)	0.371
	0.51-1.50	18 (42)	52 (54)	
	> 1.50	8 (19)	12 (12)	
Median FIB4 score (IQR)		1.77 (1.19–3.22)	2.29 (1.58–3.27)	0.179
FIB4 level (%)				
	≤ 1.45	16 (37)	22 (23)	0.199
	1.46-3.25	17 (40)	50 (51)	
	> 3.25	10 (23)	25 (26)	
DAA regimen (%)				
	SOF+RBV	-	1 (1)	
	SOF+DCV+RBV	24 (56)	62 (59)	< 0.001
	SOF+DCV	19 (44)	8 (8)	
	SOF+SMV+RBV	-	12 (11)	
	SOF+SMV	-	7 (7)	
	3D+RBV	-	3 (3)	
	3D	-	12 (11)	
Treatment duration (%)				
	12 weeks	38 (88)	92 (88)	0.271
	24 weeks	5 (12)	8 (7)	
	UNK	-	5 (5)	

IQR = interquartile range; Peg-IFN = Pegylated interferon; THE = transient hepatic elastography; APRI = AST to platelet ratio index; FIB-4 = fibrosis 4 score; DAA = direct acting antiviral; SOF = sofosbuvir; RBV = ribavirin; DCV = daclatasvir; SMV = simeprevir; 3D = Veruprevir + ritonavir + ombitasvir + dasabuvir; UNK = unknown.

The co-infection individuals had more variation in genotype than the mono-infection group. Regarding genotype distribution, in the mono-infection group, 90 participants (71%) were genotype 1, one participant was genotype 2, 12 participants (80%) were genotype 3, and two did not have genotype information available. In the co-infection group, 37 participants (29%) were genotype 1, one was genotype 2, three (20%) were genotype 3 and four were genotype 4.

### Post-treatment change in liver fibrosis

Data on APRI and FIB-4 scores were available for 140 participants before treatment onset, 118 participants at six months, and 119 participants at 12 months after treatment completion. Data on pre- and post-treatment THE were available for all participants, by definition of study enrollment. Table 2 median change in liver fibrosis scores. The median interval between elastography and treatment initiation was 360 days, with the 25^th^ percentile at 220 days and the 75^th^ percentile at 629 days interval.

**Table 2:** Change in liver fibrosis scores (pre-treatment minus six months post-treatment) by group

	HIV-HCV N = 43	HCV N = 105	p-value
Median change in THE (IQR)	0.8 (−0.6 to 4.5)	2.7 (0.7 to 5.6)	0.029
Median change in APRI (IQR)	0.20 (0.09 to 0.72)	0.31 (0.17 to 0.79)	0.299
Median change in FIB4 (IQR)	0.18 (−0.11 to 0.72)	0.63 (0 to 1.45)	0.129

IQR = interquartile range; THE = transient hepatic elastography, in kilopascals; APRI = AST to platelet ratio index; FIB-4 = Fibrosis 4 score.

**Figure 1:**
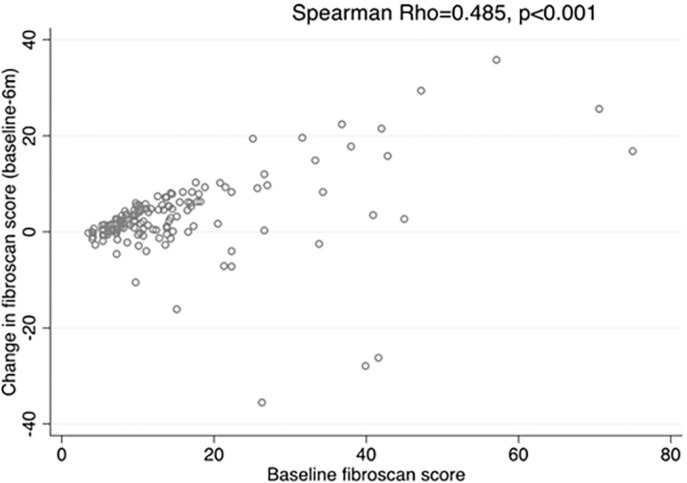
Correlation between baseline THE score and change in THE at 6m.

At six months post-treatment, the median reductions observed in the THE, APRI, and FIB-4 were 2.7 kPa, 0.31 units, and 0.63 units for participants in the chronic HCV infection group, and 0.8 kPa, 0.2 units, and 0.18 units for participants in the HIV-HCV co-infection group (p-values 0.029, 0.299, and 0.129, respectively). We found a statistically significant, positive correlation between pre-treatment THE values and reduction in the THE scores (Spearman Rho 0.485, p < 0.001; Figure 1 ), suggesting higher improvement in liver fibrosis for participants with higher baseline THE measurements. There were no statistically significant differences in APRI or FIB-4 comparing measurements taken at six and 12 months post-treatment (p = 0.123 and p = 0.561, respectively).

### Associations between HIV co-infection and change in THE scores

Table 3 shows the results of a linear regression model addressing factors associated with changes in the THE scores following HCV treatment.

**Table 3:** Univariable and multivariable models of factors associated with change in the THE score

		Unadjusted coefficients (95%CI)	Adjusted coefficients (95%CI)	p-values for adjusted coefficients
Group				
	HCV	Reference	Reference	-
	HIV-HCV	−1.86 (−5.24 to 1.51)	−1.82 (−4.90 to 1.26)	0.245
Age category, years				
	< 49.5	Reference	Reference	-
	49.5–56.9	−0.04 (−4.30 to 4.21)	0.14 (−3.77 to 4.05)	0.945
	57–65.9	1.65 (−2.43 to 5.73)	0.40 (−3.27 to 4.08)	0.829
	≥ 66	−1.25 (−4.88 to 2.38)	−1.96 (−5.17 to 1.24)	0.228
Sex				
	Male	Reference	Reference	-
	Female	2.03 (−0.61 to 4.67)	1.90 (−0.53 to 4.34)	0.124
Pre-treatment THE (per unit increase)		0.27 (0.09 to 0.45)	0.26 (0.08 to 0.45)	0.005

CI = confidence interval; THE = transient hepatic elastography.

Compared with HCV infection alone, co-infection with HIV was not significantly associated with the outcome on either the univariable model or in the model adjusted for age, sex, and pre-treatment THE. Higher pre-treatment THE scores were associated with higher change in THE; for each unit increase in pre-treatment THE, the model predicted that the reduction in THE was on average 0.26 higher (95%CI 0.08 to 0.45), adjusted age categories, sex, and co-infection group.

## DISCUSSION

We included participants with chronic HCV infection with or without HIV co-infection to investigate differences in liver fibrosis reduction following treatment with DAAs. Although the median reduction in liver fibrosis as measured by the THE score was higher in the HCV infection group, HIV co-infection was not significantly associated with the reduction in THE scores in regression models. In contrast, higher pre-treatment THE was associated with significantly higher THE reduction. Our findings suggest no strong impact of HIV co-infection on the reduction of liver fibrosis following HCV treatment. Moreover, our findings suggest that the benefits of HCV treatment concerning reduction of liver fibrosis may be greater for people with higher pre-treatment fibrosis

Patients who achieve SVR after successful treatment with DAAs often display significant improvements in liver function, which is likely related to a reduction in liver stiffness, probably due to the alleviation of HCV-associated intrahepatic inflammation^25^ .

Previous literature consistently shows a reduction in elastography values post-treatment, as well as a correlation between pre-treatment values and predictions of cirrhosis decompensation^10 , 12 , 26 , 27^ . However, studies that demonstrated a correlation between pre-treatment fibrosis scores measured by elastography and greater post-treatment fibrosis reduction are scarce. Our findings confirm that participants with co-infection have similar, if not equal, levels of fibrosis regression after treatment, contrary to earlier assumptions of lower responses^28^ . This is the second study of its kind to take place in Brazil and the first in Sao Paulo. Notably, this study includes a high proportion of women, older participants, and a high proportion of people with F4 fibrosis score.

We did not find significant improvements in APRI and FIB4 results from six to 12 months post treatment analysis. Other literature has also made the same observation, which suggests that early post-treatment variation observed in APRI and FIB4 is related to changes in inflammation rather than changes in fibrosis^29^ . This observation in the first year after successful HCV treatment may be due to the stabilization of hepatic inflammation and the slower process of true fibrosis regression.

A recent study on a similar population with co-infection used a longer-term approach, following participants and measuring fibrosis scores for four years after treatment. The study found a substantial decrease in fibrosis in the first year post-treatment, followed by stabilization of these parameters throughout the remainder of the period. These findings highlight the importance of closer follow-up in these patients to monitor potential new episodes of liver disease progression and further support the need for ongoing surveillance after successful treatment^30^ .

Limitations of this study include significant baseline demographic differences between the HCV mono-infection and HCV-HIV co-infection groups. We attempted to control this by using an adjusted analysis. The co-infection group was predominantly composed of participants assigned male at birth, younger individuals, and those with lower initial fibrosis scores, as measured by THE. These baseline differences may introduce bias, as factors such as age, sex, and initial fibrosis severity are can confound fibrosis regression outcomes. However, a recent study in a large cohort of participants with mono and co-infection found that liver fibrosis regression was independent of sex^31^ . Another possible bias at this cohort is the lack of information regarding body mass index of the participants. This is a potentially important issue since liver elastography may overestimate fibrosis in people with abundant subcutaneous abdominal fat^31 – 33^ .

A significant percentage of participants in both groups were categorized as F4 stage prior to treatment onset, suggesting established cirrhosis. The F4 stage has a lower bound at 12.5 kPa but no upper limit, therefore admitting wider variations which could result in inaccuracies of THE estimates. The study showed liver stiffness regression, but we were unable to follow-up the participants to look for other signs of liver disease progression and or episodes of cirrhosis decompensations, we also could not obtain data on Controlled Attenuation Parameters (CAP). Finally, this was a single-site study conducted at a reference tertiary clinic, which could limit the generalizability of our findings, and we included a modest samples size, particularly for the HIV-HCV co-infection group. Our study may have been underpowered to show small effects of the HIV co-infection on post-treatment reduction of liver fibrosis. This study may not be generalizable to patients outside of urban settings, or in places where DAA access is expensive or limited.

The study's strengths include the large number of female participants, no significant differences between the groups in terms of genotypes, previous IFN + RBV treatments, DAA treatment duration, or APRI and FIB-4 values. Notably, improvements were still observed despite a high prevalence of cirrhosis among participants. Additionally, we made efforts to include all the available patients who attended within this service, only excluding patients with incomplete records.

Our results are reassuring in demonstrating that the attenuation of liver fibrosis following HCV treatment with DAAs is not strongly impacted by HIV co-infection. Along with the established understanding that HIV can accelerate HCV disease progression and its detrimental outcomes^34^ , our findings support the systematic investigation of chronic HCV infection in people with HIV, and the indication of treatment with DAAs for this population.

## CONCLUSION

We found no strong impact of HIV co-infection in the reduction of liver fibrosis following HCV treatment with DAAs, whereas higher pre-treatment THE was associated with significantly higher THE reduction. Our results support the administration of HCV treatment to people with chronic HCV infection, including those with HIV co-infection and those with higher pre-treatment liver fibrosis. They also support that successful treatment results in measurable improvements within 12 months in fibrosis scores. This suggests that the earlier this treatment is done, the higher fibrosis regression this population will achieve. This observational data shows that people living with HCV and living with HIV-HCV co-infection all benefit from DAA therapy.

DATA AVAILABILITY

The complete anonymized dataset supporting the findings of this study is available from https://doi.org/10.48331/SCIELODATA.399ANF
